# 2-Isopropyl-4-meth­oxy-5-methyl­phenyl benzoate

**DOI:** 10.1107/S160053681000930X

**Published:** 2010-03-17

**Authors:** Mohamed Moumou, Mohamed Akssira, Ahmed Elhakmaoui, Lahcen El Ammari, Ahmed Benharref, Moha Berraho

**Affiliations:** a Laboratoire de Chimie Bioorganique et Analytique, URAC 22, Faculté des Sciences et Techniques, 20800 Mohammedia, Morocco; b Laboratoire de Chimie du Solide Appliquée, Faculté des Sciences, Avenue Ibn Battouta BP 1014 Rabat, Morocco; c Laboratoire de Chimie Biomolécules, Substances Naturelles et Réactivité, URAC 16, Faculté des Sciences Semlalia, BP 2390 Bd My abdellah, 40000 Marrakech, Morocco

## Abstract

The title compound, C_18_H_20_O_3_, a hemisynthetic product, was obtained by the reaction of benzoyl chloride and *p*-methoxy­thymol. The structure comprises two benzene rings bridged by a carboxyl group; the dihedral angle between the rings is 73.54 (8)°.

## Related literature

For background to the phytochemical study of Moroccan plants, see: Barrero *et al.* (2005 ▶); Zrira *et al.* (2005 ▶). For background to the medicinal inter­est in *Tetra­clinis artculata*, from which the title compound was extracted, see: Aitigri *et al.* (1990 ▶).
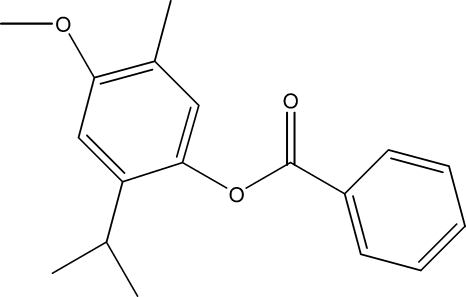


## Experimental

### 

#### Crystal data

C_18_H_20_O_3_
*M* *_r_* = 284.34
Monoclinic, 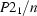

*a* = 8.4765 (4) Å
*b* = 8.0880 (4) Å
*c* = 23.8119 (11) Å
β = 98.202 (2)°
*V* = 1615.80 (13) Å^3^
*Z* = 4
Mo *K*α radiation
μ = 0.08 mm^−1^
*T* = 298 K
0.27 × 0.17 × 0.12 mm

#### Data collection

Bruker X8 APEX CCD area-detector diffractometer
14992 measured reflections
2912 independent reflections
2302 reflections with *I* > 2σ(*I*)
*R*_int_ = 0.028

#### Refinement

*R*[*F* ^2^ > 2σ(*F* ^2^)] = 0.042
*wR*(*F* ^2^) = 0.119
*S* = 1.02
2912 reflections
194 parameters
H-atom parameters constrained
Δρ_max_ = 0.18 e Å^−3^
Δρ_min_ = −0.18 e Å^−3^

### 

Data collection: *APEX2* (Bruker, 2009 ▶); cell refinement: *SAINT-Plus* (Bruker, 2009 ▶); data reduction: *SAINT-Plus*; program(s) used to solve structure: *SHELXS97* (Sheldrick, 2008 ▶); program(s) used to refine structure: *SHELXL97* (Sheldrick, 2008 ▶); molecular graphics: *ORTEP-3 for Windows* (Farrugia, 1997 ▶); software used to prepare material for publication: *WinGX* (Farrugia, 1999 ▶).

## Supplementary Material

Crystal structure: contains datablocks I, global. DOI: 10.1107/S160053681000930X/tk2638sup1.cif

Structure factors: contains datablocks I. DOI: 10.1107/S160053681000930X/tk2638Isup2.hkl

Additional supplementary materials:  crystallographic information; 3D view; checkCIF report

## Figures and Tables

**Fig. 1.**
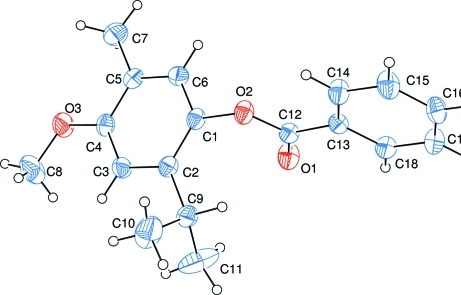
The molecular structure of (I), showing the atom-labelling scheme. Displacement ellipsoids are drawn at the 30% probability level. H atoms are represented as small spheres of arbitrary radii.

## supplementary crystallographic information

### Comment

The title compound, (I), was investigated as a part of our study of essential oils isolated from the sawdust of *Tetraclinis articulata* originating from the region of Essaouira in Morocco (Aitigri *et al.*, 1990; Barrero *et al.*, 2005; Zrira *et al.*, 2005). In this paper, we present the crystal structure of (I), which was synthesised by the reaction of the benzoyl chloride and *p*-methoxythymol (see Experimental). The molecular structure of (I), Fig. 1, shows the two benzene rings are almost perpendicular with the dihedral angle between them being 73.54 (8)°.

### Experimental

The *Tetraclinis articulata* (Vahl) Masters was collected in the region of Essaouira (Morocco). Wood sawdust was hydro-distilled in a Clevenger-type apparatus for 6 h to produce essential oils in 3% yield. The oil was then extracted by diethylether, dried over Mg_2_SO_4_, and the solvent evaporated. The oil was then subjected to silica gel column chromatography by eluting with hexane-ethyl acetate (98:2). The fifth fraction contained *p*-methoxythymol as the major compound. The structure of this product was confirmed by reaction with benzoyl chloride (0.74 g, 5.3 mmol) and crude fifth fraction (0.8 g) in a solution of 10% NaOH (50 ml). The mixture was left under agitation at 298 K for 1 h. The resulting crystalline precipitate was filtered and recrystallized from methanol. The air-dried crystal (0.7 g) had a melting point of 259–260 K.

### Refinement

All H atoms were fixed geometrically and treated as riding with C—H = 0.93 Å (aromatic), 0.96 Å (methyl), and 0.98 Å (methine), and with U_iso_(H) = 1.2U_eq_(aromatic, methine) or U_iso_(H) = 1.5U_eq_(methyl).

### Crystal data

**Table:**

C_18_H_20_O_3_	*F*(000) = 608
*M**_r_* = 284.34	*D*_x_ = 1.169 Mg m^−^^3^
Monoclinic, *P*2_1_/*n*	Mo *K*α radiation, λ = 0.71073 Å
Hall symbol: -P 2yn	Cell parameters from 14992 reflections
*a* = 8.4765 (4) Å	θ = 2.5–25.2°
*b* = 8.0880 (4) Å	µ = 0.08 mm^−^^1^
*c* = 23.8119 (11) Å	*T* = 298 K
β = 98.202 (2)°	Prism, colourless
*V* = 1615.80 (13) Å^3^	0.27 × 0.17 × 0.12 mm
*Z* = 4	

### Data collection

**Table:**

Bruker X8 APEX CCD area-detector diffractometer	2302 reflections with *I* > 2σ(*I*)
Radiation source: fine-focus sealed tube	*R*_int_ = 0.028
graphite	θ_max_ = 25.2°, θ_min_ = 2.5°
φ and ω scans	*h* = −10→10
14992 measured reflections	*k* = −9→9
2912 independent reflections	*l* = −28→28

### Refinement

**Table:**

Refinement on *F*^2^	Primary atom site location: structure-invariant direct methods
Least-squares matrix: full	Secondary atom site location: difference Fourier map
*R*[*F*^2^ > 2σ(*F*^2^)] = 0.042	Hydrogen site location: inferred from neighbouring sites
*wR*(*F*^2^) = 0.119	H-atom parameters constrained
*S* = 1.02	*w* = 1/[σ^2^(*F*_o_^2^) + (0.0574*P*)^2^ + 0.3991*P*] where *P* = (*F*_o_^2^ + 2*F*_c_^2^)/3
2912 reflections	(Δ/σ)_max_ < 0.001
194 parameters	Δρ_max_ = 0.18 e Å^−^^3^
0 restraints	Δρ_min_ = −0.18 e Å^−^^3^

### Fractional atomic coordinates and isotropic or equivalent isotropic displacement parameters (Å^2^)

**Table:**

	*x*	*y*	*z*	*U*_iso_*/*U*_eq_	
C1	0.77989 (18)	0.64673 (18)	0.08713 (6)	0.0432 (4)	
C2	0.69644 (17)	0.56847 (18)	0.12520 (6)	0.0430 (4)	
C3	0.62554 (18)	0.41749 (19)	0.10781 (6)	0.0474 (4)	
H3	0.5675	0.3612	0.1321	0.057*	
C4	0.63938 (18)	0.34959 (19)	0.05553 (7)	0.0467 (4)	
C5	0.72412 (18)	0.4313 (2)	0.01763 (6)	0.0471 (4)	
C6	0.79271 (19)	0.5809 (2)	0.03449 (6)	0.0483 (4)	
H6	0.8487	0.6387	0.0099	0.058*	
C7	0.7392 (3)	0.3562 (3)	−0.03917 (7)	0.0707 (5)	
H7A	0.7937	0.4320	−0.0608	0.106*	
H7B	0.6349	0.3338	−0.0592	0.106*	
H7C	0.7985	0.2550	−0.0338	0.106*	
C8	0.4850 (3)	0.1124 (3)	0.07224 (11)	0.0960 (8)	
H8A	0.5503	0.0867	0.1075	0.144*	
H8B	0.4464	0.0117	0.0538	0.144*	
H8C	0.3963	0.1789	0.0795	0.144*	
C9	0.6813 (2)	0.6391 (2)	0.18320 (6)	0.0508 (4)	
H9	0.7425	0.7423	0.1867	0.061*	
C10	0.5123 (3)	0.6843 (4)	0.18816 (10)	0.0985 (9)	
H10A	0.4737	0.7620	0.1589	0.148*	
H10B	0.5078	0.7332	0.2246	0.148*	
H10C	0.4472	0.5868	0.1841	0.148*	
C11	0.7565 (4)	0.5276 (3)	0.22999 (9)	0.1153 (10)	
H11A	0.6928	0.4298	0.2311	0.173*	
H11B	0.7631	0.5845	0.2656	0.173*	
H11C	0.8616	0.4973	0.2232	0.173*	
C12	0.99177 (17)	0.80615 (17)	0.13445 (6)	0.0416 (3)	
C13	1.05364 (17)	0.97651 (17)	0.14447 (6)	0.0402 (3)	
C14	0.9796 (2)	1.11338 (19)	0.11761 (7)	0.0535 (4)	
H14	0.8884	1.1002	0.0912	0.064*	
C15	1.0407 (2)	1.2690 (2)	0.12988 (8)	0.0605 (5)	
H15	0.9906	1.3607	0.1118	0.073*	
C16	1.1752 (2)	1.2895 (2)	0.16867 (8)	0.0561 (4)	
H16	1.2156	1.3950	0.1769	0.067*	
C17	1.2503 (2)	1.1547 (2)	0.19528 (8)	0.0561 (4)	
H17	1.3419	1.1688	0.2214	0.067*	
C18	1.19010 (18)	0.99813 (19)	0.18335 (7)	0.0478 (4)	
H18	1.2412	0.9069	0.2014	0.057*	
O1	1.05685 (14)	0.68399 (13)	0.15440 (5)	0.0593 (3)	
O2	0.85023 (13)	0.80333 (12)	0.10003 (5)	0.0495 (3)	
O3	0.57625 (15)	0.20040 (15)	0.03683 (5)	0.0644 (4)	

### Atomic displacement parameters (Å^2^)

**Table:**

	*U*^11^	*U*^22^	*U*^33^	*U*^12^	*U*^13^	*U*^23^
C1	0.0463 (8)	0.0325 (8)	0.0480 (8)	−0.0015 (6)	−0.0032 (7)	0.0000 (6)
C2	0.0447 (8)	0.0382 (8)	0.0449 (8)	0.0017 (6)	0.0021 (6)	−0.0034 (6)
C3	0.0504 (9)	0.0436 (9)	0.0490 (9)	−0.0072 (7)	0.0095 (7)	−0.0038 (7)
C4	0.0465 (8)	0.0400 (8)	0.0523 (9)	−0.0046 (7)	0.0025 (7)	−0.0093 (7)
C5	0.0486 (9)	0.0496 (9)	0.0416 (8)	−0.0003 (7)	0.0011 (7)	−0.0046 (7)
C6	0.0525 (9)	0.0475 (9)	0.0440 (8)	−0.0030 (7)	0.0035 (7)	0.0057 (7)
C7	0.0856 (14)	0.0759 (13)	0.0517 (10)	−0.0091 (11)	0.0138 (9)	−0.0162 (9)
C8	0.123 (2)	0.0693 (14)	0.1051 (17)	−0.0538 (14)	0.0470 (15)	−0.0320 (13)
C9	0.0592 (10)	0.0455 (9)	0.0476 (9)	−0.0051 (7)	0.0069 (7)	−0.0086 (7)
C10	0.0700 (13)	0.142 (2)	0.0866 (15)	−0.0023 (14)	0.0221 (12)	−0.0558 (16)
C11	0.199 (3)	0.0939 (18)	0.0466 (11)	0.0372 (19)	−0.0033 (15)	0.0001 (11)
C12	0.0446 (8)	0.0349 (8)	0.0449 (8)	0.0028 (6)	0.0046 (6)	0.0006 (6)
C13	0.0428 (8)	0.0335 (7)	0.0449 (8)	0.0013 (6)	0.0087 (6)	0.0001 (6)
C14	0.0505 (9)	0.0384 (9)	0.0673 (10)	−0.0001 (7)	−0.0062 (8)	0.0053 (8)
C15	0.0617 (11)	0.0332 (8)	0.0836 (13)	−0.0006 (7)	−0.0003 (9)	0.0082 (8)
C16	0.0568 (10)	0.0375 (9)	0.0741 (11)	−0.0090 (7)	0.0100 (9)	−0.0060 (8)
C17	0.0490 (9)	0.0526 (10)	0.0641 (10)	−0.0059 (8)	−0.0008 (8)	−0.0078 (8)
C18	0.0485 (9)	0.0405 (9)	0.0531 (9)	0.0044 (7)	0.0030 (7)	0.0015 (7)
O1	0.0596 (7)	0.0331 (6)	0.0803 (8)	0.0040 (5)	−0.0070 (6)	0.0040 (5)
O2	0.0540 (6)	0.0319 (5)	0.0583 (7)	−0.0037 (5)	−0.0064 (5)	0.0031 (5)
O3	0.0756 (8)	0.0526 (7)	0.0670 (8)	−0.0224 (6)	0.0165 (6)	−0.0217 (6)

### Geometric parameters (Å, °)

**Table:**

C1—C2	1.380 (2)	C9—H9	0.9800
C1—C6	1.380 (2)	C10—H10A	0.9600
C1—O2	1.4149 (17)	C10—H10B	0.9600
C2—C3	1.397 (2)	C10—H10C	0.9600
C2—C9	1.517 (2)	C11—H11A	0.9600
C3—C4	1.381 (2)	C11—H11B	0.9600
C3—H3	0.9300	C11—H11C	0.9600
C4—O3	1.3685 (18)	C12—O1	1.1964 (17)
C4—C5	1.397 (2)	C12—O2	1.3531 (18)
C5—C6	1.378 (2)	C12—C13	1.481 (2)
C5—C7	1.504 (2)	C13—C14	1.384 (2)
C6—H6	0.9300	C13—C18	1.386 (2)
C7—H7A	0.9600	C14—C15	1.377 (2)
C7—H7B	0.9600	C14—H14	0.9300
C7—H7C	0.9600	C15—C16	1.371 (2)
C8—O3	1.415 (2)	C15—H15	0.9300
C8—H8A	0.9600	C16—C17	1.371 (2)
C8—H8B	0.9600	C16—H16	0.9300
C8—H8C	0.9600	C17—C18	1.379 (2)
C9—C10	1.500 (3)	C17—H17	0.9300
C9—C11	1.503 (3)	C18—H18	0.9300
			
C2—C1—C6	122.25 (14)	C9—C10—H10A	109.5
C2—C1—O2	120.49 (13)	C9—C10—H10B	109.5
C6—C1—O2	117.19 (13)	H10A—C10—H10B	109.5
C1—C2—C3	116.42 (14)	C9—C10—H10C	109.5
C1—C2—C9	122.90 (14)	H10A—C10—H10C	109.5
C3—C2—C9	120.68 (14)	H10B—C10—H10C	109.5
C4—C3—C2	121.77 (15)	C9—C11—H11A	109.5
C4—C3—H3	119.1	C9—C11—H11B	109.5
C2—C3—H3	119.1	H11A—C11—H11B	109.5
O3—C4—C3	124.36 (14)	C9—C11—H11C	109.5
O3—C4—C5	114.83 (13)	H11A—C11—H11C	109.5
C3—C4—C5	120.80 (14)	H11B—C11—H11C	109.5
C6—C5—C4	117.50 (14)	O1—C12—O2	123.04 (13)
C6—C5—C7	122.01 (15)	O1—C12—C13	124.86 (14)
C4—C5—C7	120.49 (15)	O2—C12—C13	112.10 (12)
C5—C6—C1	121.25 (14)	C14—C13—C18	119.23 (14)
C5—C6—H6	119.4	C14—C13—C12	122.88 (13)
C1—C6—H6	119.4	C18—C13—C12	117.88 (13)
C5—C7—H7A	109.5	C15—C14—C13	120.07 (15)
C5—C7—H7B	109.5	C15—C14—H14	120.0
H7A—C7—H7B	109.5	C13—C14—H14	120.0
C5—C7—H7C	109.5	C16—C15—C14	120.35 (16)
H7A—C7—H7C	109.5	C16—C15—H15	119.8
H7B—C7—H7C	109.5	C14—C15—H15	119.8
O3—C8—H8A	109.5	C15—C16—C17	120.11 (15)
O3—C8—H8B	109.5	C15—C16—H16	119.9
H8A—C8—H8B	109.5	C17—C16—H16	119.9
O3—C8—H8C	109.5	C16—C17—C18	120.07 (15)
H8A—C8—H8C	109.5	C16—C17—H17	120.0
H8B—C8—H8C	109.5	C18—C17—H17	120.0
C10—C9—C11	113.4 (2)	C17—C18—C13	120.16 (14)
C10—C9—C2	111.69 (14)	C17—C18—H18	119.9
C11—C9—C2	111.55 (15)	C13—C18—H18	119.9
C10—C9—H9	106.6	C12—O2—C1	117.16 (11)
C11—C9—H9	106.6	C4—O3—C8	118.13 (13)
C2—C9—H9	106.6		
